# Network pharmacology analysis and molecular docking to unveil the potential mechanisms of San-Huang-Chai-Zhu formula treating cholestasis

**DOI:** 10.1371/journal.pone.0264398

**Published:** 2022-02-23

**Authors:** Binbin Liu, Jie Zhang, Lu Shao, Jiaming Yao

**Affiliations:** Department of Digestion, Hangzhou TCM Hospital Affiliated to Zhejiang Chinese Medical University (Hangzhou Hospital of Traditional Chinese Medicine), Hangzhou, Zhejiang, China; Texas A&M University, UNITED STATES

## Abstract

**Objective:**

Chinese medicine formulae possess the potential for cholestasis treatment. This study aimed to explore the underlying mechanisms of San-Huang-Chai-Zhu formula (SHCZF) against cholestasis.

**Methods:**

The major chemical compounds of SHCZF were identified by high-performance liquid chromatography. The bioactive compounds and targets of SHCZF, and cholestasis-related targets were obtained from public databases. Intersected targets of SHCZF and cholestasis were visualized by Venn diagram. The protein-protein interaction and compound-target networks were established by Cytoscape according to the STRING database. The biological functions and pathways of potential targets were characterized by Gene Ontology and Kyoto Encyclopedia of Genes and Genomes enrichment analysis. The biological process-target-pathway network was constructed by Cytoscape. Finally, the interactions between biological compounds and hub target proteins were validated via molecular docking.

**Results:**

There 7 major chemical compounds in SHCZF. A total of 141 bioactive compounds and 83 potential targets were screened for SHCZF against cholestasis. The process of SHCZF against cholestasis was mainly involved in AGE-RAGE signaling pathway in diabetic complications, fluid shear stress and atherosclerosis, and drug metabolism-cytochrome P450. ALB, IL6, AKT1, TP53, TNF, MAPK3, APOE, IL1B, PPARG, and PPARA were the top 10 hub targets. Molecular docking showed that bioactive compounds of SHCZF had a good binding affinity with hub targets.

**Conclusions:**

This study predicted that the mechanisms of SHCZF against cholestasis mainly involved in AGE-RAGE signaling pathway in diabetic complications, fluid shear stress and atherosclerosis, and drug metabolism-cytochrome P450. Moreover, APOE, AKT1, and TP53 were the critical hub targets for bioactive compounds of SHCZF.

## Introduction

Cholestasis is a common clinical manifestation of liver disease mainly derived from the reduction or obstruction of bile flow [1]. The long-term cholestasis in liver can lead to hepatocyte dysfunctions, thereby causing severe liver diseases such as primary biliary cirrhosis, primary sclerosing cholangitis and secondary sclerosing cholangitis [2]. At present, although some drugs, such as rosiglitazone, obeticholic acid, and ursodeoxycholic acid, have been developed for cholestasis treatment, the therapeutic effect is still limited and may contribute to pruritus, dyslipidemia, and gastrointestinal symptoms [3, 4]. Therefore, the discovery of effective drugs for cholestasis treatment is of great significance.

Accumulating evidence indicated that Chinese medicines exert beneficial therapeutic effects in liver diseases and cholestasis [5, 6]. San-Huang-Chai-Zhu formula (SHCZF) is a Chinese herbal formula, which consists of five herbs, namely, Dahuang (*Rhei Radix Et Rhizome*), Huangbai (*Phellodendri Chinrnsis Cortex*), Huangzhizi (*Gardeniae Fructus*), Chaihu (*Radix Bupleuri*), and Baizhu (*Atractylodes Macrocephala Koidz*.). Previous studies indicated that these five herbs all possess the hepatoprotective effect on liver diseases. Cao et al. [7] reported that Dahuang had extensive pharmacological effects in hepatoprotective, anti-inflammatory, anticancer and so on. Huangbai and Huangzhizi were widely used to ameliorate inflammation and hepatotoxicity as a core component of herbal formula [8, 9]. Saikosaponins extracted from Chaihu showed valuable pharmacological activities of anti-inflammatory and liver protection [10]. Baizhu in Xiaoyao San formula was also validated its pharmacological effects of hepatoprotection [11]. However, the underlying pharmacological mechanism of SHCZF against cholestasis is still illusive.

Network pharmacology is a favorable method to reveal the pharmacological mechanism of Chinese medicine formulae against specific diseases and identify the relevant drugs, targets, and pathways [12–14]. This approach comprehensively investigates the interactions of bioactive ingredients, targets, and diseases, and the relationship are visualized by interaction networks. For instance, by combining the network pharmacology with the pathological examination, Xiaoyaosan decoction was proved the therapeutic effects on alleviating liver fibrosis [15]. The potential biological mechanisms of GegenQinlian decoction also were unveiled to improve insulin resistance in liver, adipose, and muscle tissue by network pharmacology analysis [16]. Therefore, network pharmacology is a commendable approach for exploring the underlying mechanisms of SHCZF against cholestasis.

In this article, the underlying mechanisms of SHCZF against cholestasis were uncovered by identifying bioactive compounds and potential target genes. Moreover, the interactions between major bioactive compounds and hub target proteins were validated by molecular docking. This study provides an essential foundation for further experimental investigations and clinical application of SHCZF against cholestasis.

## Methods

### Main ingredients analysis of SHCZF

SHCZF was prepared by mixing five herbs (Dahuang, Huangbai, Huangzhizi, Chaihu, and Baizhu) in the ratio of 4:4:3:3:4. The extract of SHCZF was obtained by adding 10 times the amount of water, soaking for 30 min, and boiling for 1.5 h. After filtering out the liquid, samples were added 8 times the amount of water and decocted for 0.5 h after boiling. Then, the obtained extract was concentrated into 2 g/mL for high-performance liquid chromatography (HPLC) determination. Samples were analyzed using a LC-20AT HPLC system (Shimadzu, Japan) and separated using an Extend-C18 column (250 mm × 4.6 mm, 5 μm) (Agilent, CA, USA) with a mobile phase consisting of 0.1% phosphoric acid (A) and acetonitrile (B). The elution gradient was as follows: 0–10 min with 90% A and 10% B, 10–20 min with 30% A and 30% B, 20–30 min with 40% A and 60% B, 30–53 min with 30% A and 70% B, 53–54 min with 90% A and 10% B, and 59 min controller stop. The molecular structures of these seven compounds of SHCZF were downloaded from ZINC (https://zinc15.docking.org/) [17].

### Screening for bioactive ingredients and targets of SHCZF

All ingredients from 5 herbs of SHCZF were retrieved from the traditional Chinese medicine integrated database (TCMID, http://www.megabionet.org/tcmid/) [18], the traditional Chinese medicine systems pharmacology database and analysis platform (TCMSP, https://old.tcmsp-e.com/tcmsp.php) [19], and herb ingredients’ targets (HIT, http://lifecenter.sgst.cn/hit/) database [20]. Totally, 227 compounds were obtained after eliminating those compounds without targets. In addition, Search tool for interacting chemicals (STITCH, http://stitch.embl.de/) database [21] and the above data sources were used to retrieve targets associated with 227 compounds from SHCZF with a setting of minimum required interaction score = 0.400 in STITCH. A total of 5216 targets was collected and the Gene ID of these targets was normalized by National Center for Biotechnology Information (NCBI) database (https://www.ncbi.nlm.nih.gov/).

### Drug-likeness calculation of SHCZF compounds

The 227 compounds of SHCZF were screened by drug-likeness evaluation. The assessment of drug-likeness properties is mainly determined by absorption, distribution, metabolism, and elimination (ADME) features of compounds [22]. The quantitative estimate of drug-likeness (*QED*) value is an important parameter to assess ADME characteristics. In this work, we calculated *QED* value described by Bickerton [23] to screen pharmaceutically active compounds in SHCZF. The equation of *QED* calculation was shown as follows:

$$QED=\exp(\frac{1}{n}\sum_{i=1}^{n}\ln d_{i})$$

In this equation, desirability functions (*d*) were obtained by integrating 8 physicochemical properties of compounds, including molecular weight (MW), the number of hydrogen bond acceptors (HBAs), the number of hydrogen bond donors (HBDs), the octanol-water partition coefficient (ALogP), the number of rotatable bonds (ROTBs), the number of aromatic rings (AROMs), molecular polar surface area (PSA), and the number of structural alerts (ALERTS). Compounds in SHCZF with *QED* ≥ 0.2 referring to the DrugBank database (https://go.drugbank.com/) were included for following analyses.

### Target selection of active compounds in SHCZF

To precisely define compound-target interaction, the enrichment scoring algorithm based on a binomial statistical model was used to screen core targets of compounds [24, 25]. The target that interacts with most of active compounds can be considered as a core target of SHCZF. The probability of being a core target was calculated as follows:

$$P_{i}(X\geq k)=\sum_{m=k}^{n}C_{n}^{m}{\left(p\right)}^{m}{\left(1-p\right)}^{n-m}$$

where, *n* is the total number of compounds in SHCZF, *p* is the ratio of the average number of compounds simultaneously interacting with the same target in the total target of SHCZF compounds, and *P*_*i*_ (*X* ≥ *k*) represents the probability of a target gene (*i*) simultaneously interacting with more than *k* active compounds. The investigated target with *P* < 0.01 can be regarded as a core target for SHCZF compounds.

### Screening of targets associated with cholestasis

The cholestasis-related targets were retrieved from the GeneCards database (https://www.genecards.org/) [26, 27], the online mendelian inheritance in man (OMIM, https://www.omim.org/) database [28], and the DisGeNET database (https://www.disgenet.org/home/) [29]. Accordingly, 56, 28, and 420 cholestasis-related targets were collected from GeneCards, OMIM, and DisGeNET databases, respectively. A total of 449 targets was obtained after removing duplicates (S1 Table).

### Construction of protein-protein interaction (PPI) network and compound-target (C-T) network

The intersection targets of SHCZF and cholestasis were visualized by a Venn diagram. PPI network of common target proteins was established and analyzed using Search Tool for the Retrieval of Interacting Genes/Proteins (STRING) dataset (https://string-db.org) [30], where each node in the network represented a target, and the node with higher degree means the more important target in the network. The C-T network of SHCZF against cholestasis was constructed using Cytoscape (v3.8.2) [31].

### Biological function enrichment analyses

In order to further explore the biological functions of SHCZF acting on cholestasis, core targets were integrated for Gene Ontology (GO) and Kyoto Encyclopedia of Genes and Genomes (KEGG) enrichment analyses [32]. GO enrichment analysis included molecular function (MF), biological process (BP), and cellular component (CC) analyses according to the GO database. KEGG enrichment analysis were performed according to the KEGG database. A hypergeometric distribution model was used to assess whether the core target genes were significantly related to specific GO terms and KEGG pathways [33], showed as follows:

$$P=1-\sum_{i=0}^{k-1}\frac{\left(\begin{array}{c} M\\ i \end{array})(\begin{array}{c} N-M\\ n-i \end{array}\right)}{\left(\begin{array}{c} N\\ n \end{array}\right)}$$

where, *N* is the total number of genes, *M* is the number of genes annotated in GO and KEGG databases, *n* is the number of investigated target genes of SHCZF, and *k* is the number of intersection genes of SHCZF and annotated genes. *P*-values that corrected by the Bonferroni method reflected the relevance between potential targets and GO terms or KEGG pathways. GO terms and KEGG pathways with *P*-value < 0.01 were considered as significant relevance. Bubble charts and histograms were drawn based on the cluster profiler package R 3.15.4.

### Construction of a target-pathway network for SHCZF against cholestasis

To elucidate the pharmacological mechanism of SHCZF in cholestasis treatment, Cytoscape was used to construct a BP-target-pathway network. The degree, betweenness and centeredness of potential target were calculated by a CytoHubba plugin [34]. The core targets, top 15 KEGG pathways, and top 15 BPs were included in the network. Targets with flesh-colored circles, pathways with green circles, and BPs with purple circles were presented as nodes, and the interactions between nodes were expressed as edges.

### Molecular docking

Molecular docking was conducted to validate the interactions between bioactive compounds and target proteins of SHCZF against cholestasis. The top 10 hub target proteins were selected for molecular docking and used for PPI network construction by a CytoHubba plugin in Ctytoscape. The 3D structures of target proteins were obtained from Protein Data Bank (PDB, https://www.rcsb.org/) [35]. After deleting water molecules using PyMol (v2.3.0) [36], the obtained protein structures were imported into AutoDockTools (v1.5.6) to construct mating pocket of molecular docking. Molecular docking with bioactive compounds was performed using AutoDock Vina (v1.1.2) [37] based on the data collected above.

## Results

### Major ingredients in SHCZF

HPLC was performed to identify the major chemical compounds in SHCZF. Seven main compounds of SHCZF were obtained, including chrysophanol, emodin, physcion, rhein, aloe-emodin, berberine chloride, gardenoside (S1A–S1G Fig). The chemical structures of these 7 compounds were shown in Table 1.

**Table 1:** Chemical structures of 7 major compounds of San-Huang-Chai-Zhu formula (SHCZF).

Synonyms	Cas	Molecular Formula
Chrysophanol	481-74-3	C_15_H_10_O_4_
Emodin	518-82-1	C_15_H_10_O_5_
Physcion	521-61-9	C_16_H_12_O_5_
Rhein	478-43-3	C_15_H_8_O_6_
Aloe-emodin	481-72-1	C_15_H_10_O_5_
Berberine chloride	633-65-8	C_20_H_18_ClNO_4_
Gardenoside	24512-62-7	C_17_H_24_O_11_

### Bioactive components and targets of SHCZF

*QED* is a critical indicator to evaluate the drug-likeness of compounds. According to the *QED* values, 216 drug-likeness components in SHCZF were obtained based on the TCMID, TCMSP, and HIT database. Moreover, 162 active compounds and 457 SHCZF compound-related targets were collected by combining the public databases with a binomial statistical model. There were 19, 40, 34, 93, and 22 bioactive compounds in Dahuang, Huangbai, Huangzhizi, Chaihu, and Baizhu of SHCZF, respectively (S2 Table).

### Potential targets of SHCZF active compounds for cholestasis treatment

**Fig 1:**
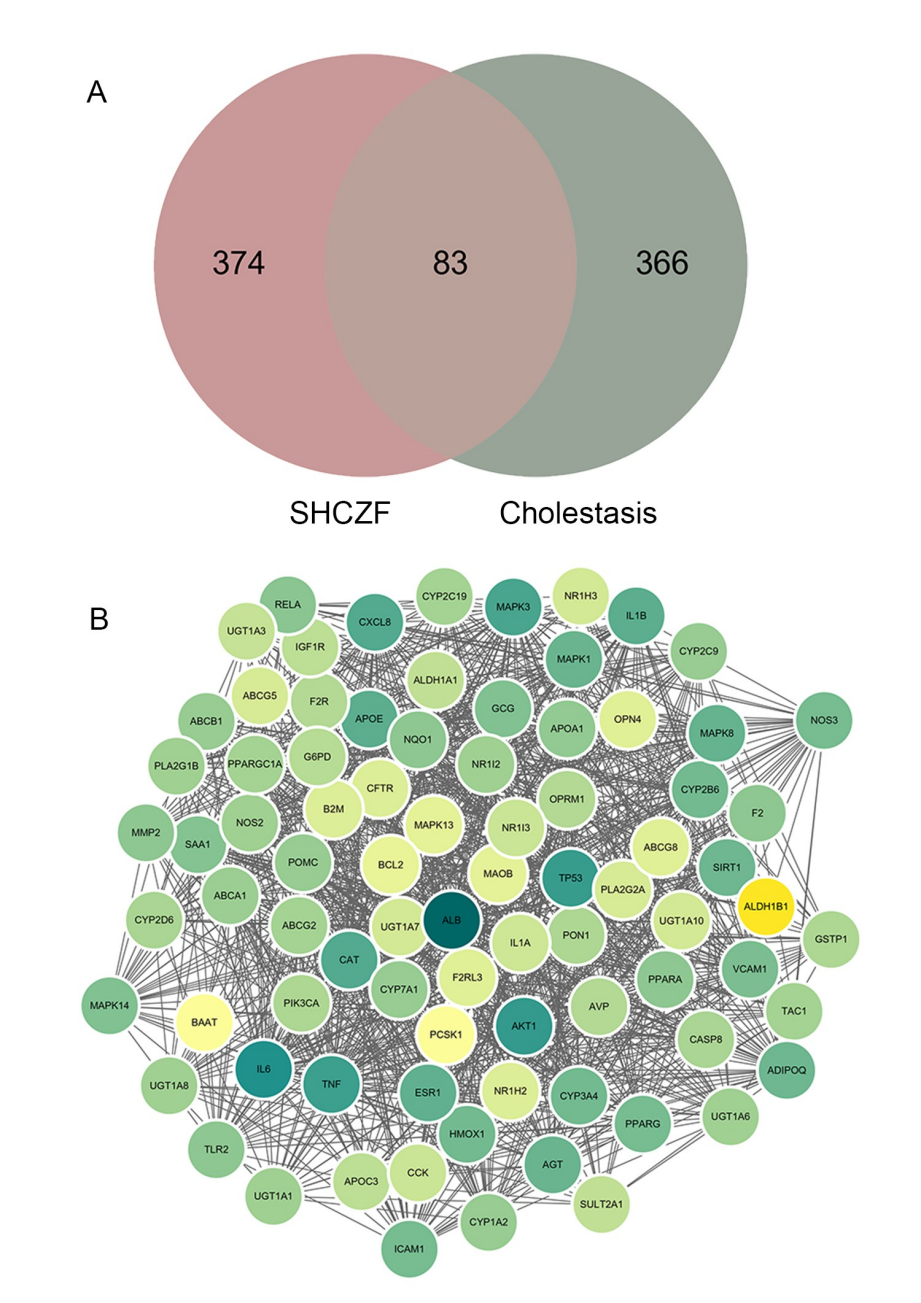
The 83 potential targets for San-Huang-Chai-Zhu formula (SHCZF) in cholestasis treatment. **(A)** Intersection of SHCZF and cholestasis targets was visualized by Venn diagram. **(B)** Protein-protein interaction (PPI) network of 83 common targets. Each node represents a common target for SHCZF and cholestasis, and each edge represents the association between two targets. The darker green means the higher degree value, and the average degree is 24.9.

According to the GeneCards, OMIM, and DisGeNET databases, a total of 449 cholestasis-related target genes were obtained after eliminating duplicates (S1 Table). The intersection between 457 SHCZF targets and 449 cholestasis-related targets was presented by a Venn diagram. As a result, there were 83 overlapping targets considered as core targets associated with both SHCZF compounds and cholestasis (Fig 1A & Table 2). Furthermore, 83 potential targets were input into the STRING database to construct a PPI network. Nodes and edges in the PPI network represent targets and protein-protein associations, respectively. The PPI network included 83 nodes and 1034 edges. Green and yellow circles in the PPI network stood for 83 potential targets. The degree of targets represents the number of links to nodes, and the target with higher degree can be regarded as the more important target. In this PPI network, the darker green circles mean the targets with higher degree and yellow circles mean less importance. The average node degree of this PPI network was 24.9, and ALB, IL6, AKT1, TP53, TNF, MAPK3, APOE, IL1B, PPARG, and PPARA were top 10 targets with high degrees (Fig 1B).

**Table 2:** 83 potential targets of SHCZF against cholestasis.

Gene ID	Target Name	Gene ID	Target Name	Gene ID	Target Name	Gene ID	Target Name
19	ABCA1	1559	CYP2C9	4846	NOS3	7157	TP53
183	AGT	1565	CYP2D6	4988	OPRM1	7376	NR1H2
207	AKT1	1576	CYP3A4	5122	PCSK1	7412	VCAM1
213	ALB	1581	CYP7A1	5243	ABCB1	8856	NR1I2
216	ALDH1A1	1728	NQO1	5290	PIK3CA	9002	F2RL3
219	ALDH1B1	2099	ESR1	5319	PLA2G1B	9370	ADIPOQ
335	APOA1	2147	F2	5320	PLA2G2A	9429	ABCG2
345	APOC3	2149	F2R	5443	POMC	9970	NR1I3
348	APOE	2539	G6PD	5444	PON1	10062	NR1H3
551	AVP	2641	GCG	5465	PPARA	10891	PPARGC1A
567	B2M	2950	GSTP1	5468	PPARG	23411	SIRT1
570	BAAT	3162	HMOX1	5594	MAPK1	54575	UGT1A10
596	BCL2	3383	ICAM1	5595	MAPK3	54576	UGT1A8
841	CASP8	3480	IGF1R	5599	MAPK8	54577	UGT1A7
847	CAT	3552	IL1A	5603	MAPK13	54578	UGT1A6
885	CCK	3553	IL1B	5970	RELA	54658	UGT1A1
1080	CFTR	3569	IL6	6288	SAA1	54659	UGT1A3
1432	MAPK14	3576	CXCL8	6822	SULT2A1	64240	ABCG5
1544	CYP1A2	4129	MAOB	6863	TAC1	64241	ABCG8
1555	CYP2B6	4313	MMP2	7097	TLR2	94233	OPN4
1557	CYP2C19	4843	NOS2	7124	TNF		

### Compound-target (C-T) network of SHCZF against cholestasis

**Fig 2:**
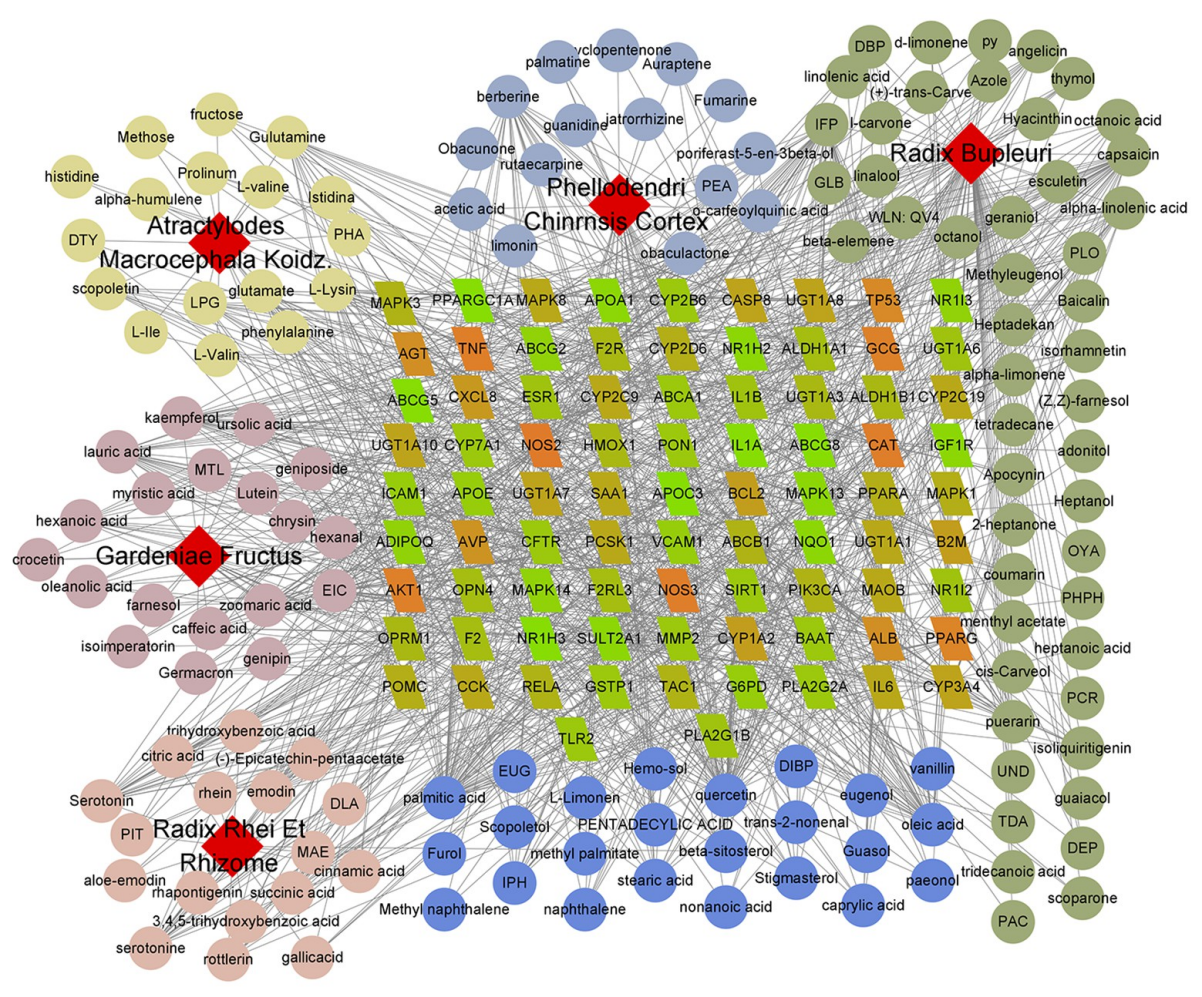
Compound-target (C-T) network of 141 bioactive compounds and 83 potential targets for SHCZF against cholestasis. There were 229 nodes in the C-T network, including 5 red diamonds for herbs from SHCZF, 83 orange (higher degree) and green (lower degree) parallelograms for potential targets, and 141 circles for bioactive compounds.

According to 83 potential targets, 141 SHCZF compounds were identified as the major ingredients acting on cholestasis (Table 3). The interactions between 83 potential targets and 141 SHCZF compounds were exhibited by a C-T network. In the C-T network, red diamonds represented 5 herbs in SHCZF, including Dahuang (*Rhei Radix Et Rhizome*), Huangzhizi (*Gardeniae Fructus*), Baizhu (*Atractylodes Macrocephala Koidz*.), Huangbai (*Phellodendri Chinrnsis Cortex*), and Chaihu (*Radix Bupleuri*). Circles with 5 different colors stood for distinct compounds from 5 herbs, among which, there were 17 compounds from *Rhei Radix Et Rhizome*, 19 from *Gardeniae Fructus*, 17 from *Atractylodes Macrocephala Koidz*., 15 from *Phellodendri Chinrnsis Cortex*, and 49 from *Radix Bupleuri*. Besides, 24 common compounds were displayed using blue circles. The parallelograms in the network represented 83 potential targets of SHCZF against cholestasis and darker orange indicated higher degree (Fig 2).

**Table 3:** 141 bioactive compounds of SHCZF against cholestasis.

Compound Name	*QED*	Compound Name	*QED*
(-)-Epicatechin-pentaacetate	0.3317	Istidina	0.4207
(+)-trans-Carveol	0.5719	jatrorrhizine	0.7352
(Z,Z)-farnesol	0.6157	kaempferol	0.6372
vanillin	0.5173	lauric acid	0.3925
2-heptanone	0.5103	l-carvone	0.5247
3,4,5-trihydroxybenzoic acid	0.4656	L-Ile	0.4718
acetic acid	0.4199	limonin	0.4519
adonitol	0.3082	linalool	0.6172
aloe-emodin	0.7330	linolenic acid	0.3326
alpha-humulene	0.4851	L-Limonene	0.4838
alpha-limonene	0.4838	L-Lysin	0.2814
alpha-linolenic acid	0.3326	LPG	0.3562
angelicin	0.4354	Lutein	0.2035
angelicin	0.4670	L-Valin	0.4120
Apocynin	0.6736	L-valine	0.4266
Auraptene	0.4124	MAE	0.4992
Azole	0.4642	menthyl acetate	0.6510
Baicalin	0.3617	Methose	0.3101
berberine	0.6633	Methyl naphthalene	0.5294
berberine	0.8245	methyl palmitate	0.2468
beta-elemene	0.5799	Methyleugenol	0.6599
beta-sitosterol	0.4354	MTL	0.2704
caffeic acid	0.4750	myristic acid	0.4490
caprylic acid	0.5818	naphthalene	0.5114
capsaicin	0.5370	nonanoic acid	0.5775
chrysin	0.8206	obaculactone	0.4519
cinnamic acid	0.6504	Obacunone	0.4784
cis-Carveol	0.5719	o-caffeoylquinic acid	0.2356
citric acid	0.4243	octanoic acid	0.5818
coumarin	0.4124	octanol	0.5480
crocetin	0.5030	oleanolic acid	0.4460
Cyclopentenone	0.4228	oleic acid	0.2030
DBP	0.4752	OYA	0.3958
DEP	0.6925	PAC	0.6684
DIBP	0.6761	paeonol	0.5478
DLA	0.4605	palmatine	0.6613
d-limonene	0.4838	palmitic acid	0.3653
DTY	0.5110	PCR	0.5390
EIC	0.2944	PEA	0.6259
emodin	0.6835	pentadecylic acid	0.4059
esculetin	0.3579	PHA	0.5664
EUG	0.6993	phenylalanine	0.5664
eugenol	0.6955	PHPH	0.5905
farnesol	0.6157	PIT	0.4834
fructose	0.3101	PLO	0.7502
Fumarine	0.7258	poriferast-5-en-3beta-ol	0.4354
Furol	0.4792	Prolinum	0.3867
gallicacid	0.4656	puerarin	0.4049
genipin	0.5093	py	0.4453
geniposide	0.2532	quercetin	0.5064
geraniol	0.6172	rhapontigenin	0.7399
Germacron	0.4329	rhein	0.7375
GLB	0.3046	rottlerin	0.2140
glutamate	0.3835	rutaecarpine	0.6889
guaiacol	0.5771	scoparone	0.5470
guanidine	0.2426	scopoletin	0.5425
Guasol	0.5771	Scopoletol	0.5425
Gulutamine	0.3835	Serotonin	0.6456
Hemo-sol	0.4838	serotonine	0.6456
Heptadekan	0.2688	stearic acid	0.3017
heptanoic acid	0.5128	Stigmasterol	0.4599
Heptanol	0.5465	succinic acid	0.5303
hexanal	0.2939	TDA	0.4900
hexanoic acid	0.5687	tetradecane	0.3217
histidine	0.4184	thymol	0.6510
Hyacinthin	0.4290	trans-2-nonenal	0.3144
IFP	0.3920	tridecanoic acid	0.4900
IPH	0.5172	trihydroxybenzoic acid	0.4656
isoimperatorin	0.4856	UND	0.4133
isoliquiritigenin	0.5824	ursolic acid	0.4433
isorhamnetin	0.6678		

### GO and KEGG enrichment analyses

**Fig 3:**
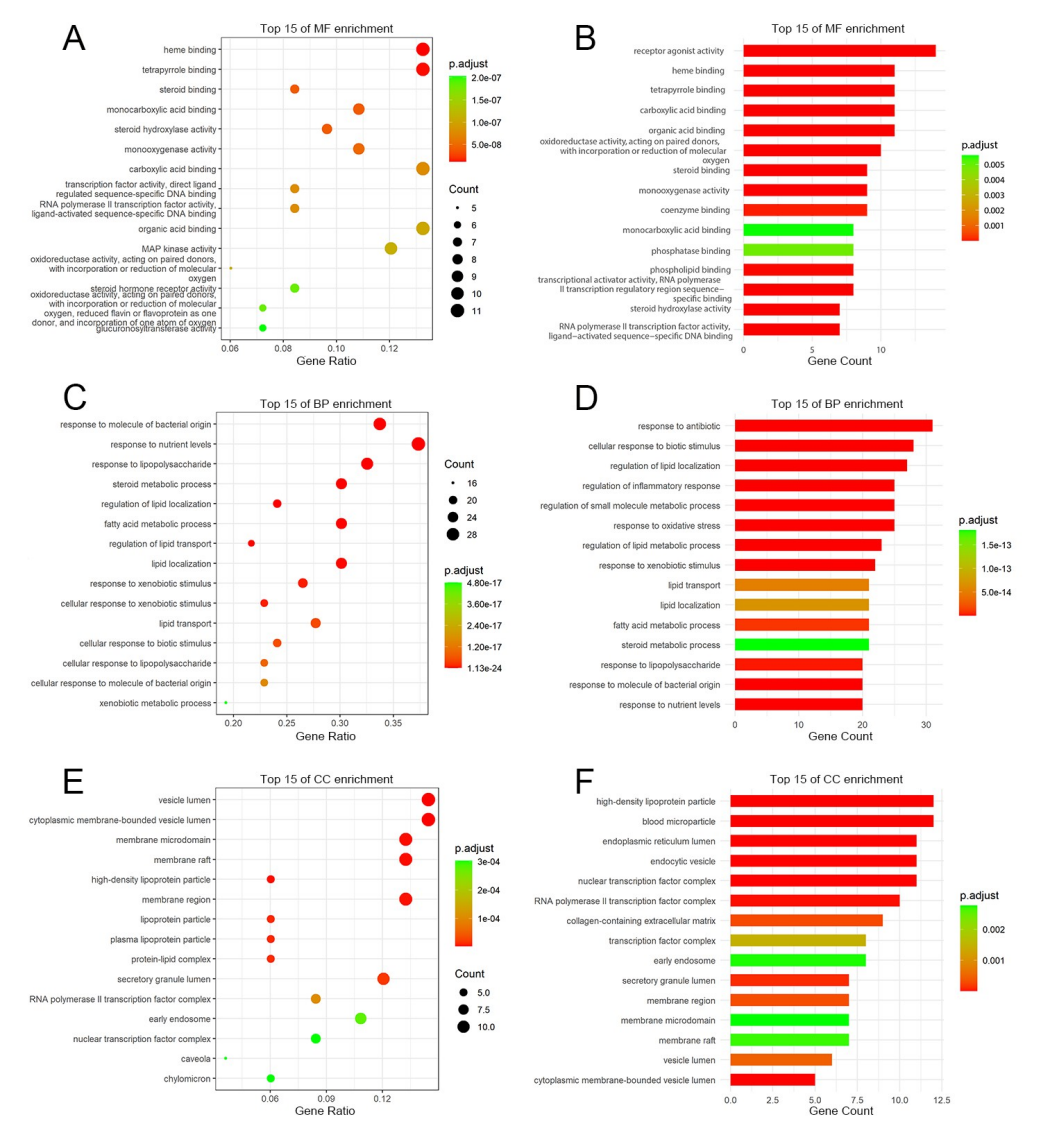
Gene Ontology (GO) enrichment analysis for 83 potential targets of SHCZF against cholestasis. **(A, B)** The bubble chart and histogram of top 15 molecular function (MF) enrichment. **(C, D)** The bubble chart and histogram of top 15 biological process (BP) enrichment. **(E, F)** The bubble chart and histogram of top 15 cellular component (CC) enrichment.

To elaborate the biological functions of 83 potential targets, targets were characterized by GO and KEGG pathway enrichment analyses. In the GO analysis, a total of 1617 GO terms were found, including 91 of MF, 1498 of BP, and 28 of CC (*p* value < 0.01). The top 15 terms of MF, BP, and CC were ranked according to the adjusted *p* value and gene count (Fig 3). Lower *p* value with red color and higher count with bigger circle indicated greater enrichment of GO terms. The bubble chart and histogram showed that MF was significantly enriched in heme binding, tetrapyrrole binding, carboxylic acid binding, receptor agonist activity, and organic acid binding, etc. (Fig 3A and 3B). The main GO terms of BP were related to response to lipopolysaccharide, regulation of lipid localization, cellular response to biotic stimulus, regulation of inflammatory response, and response to oxidative stress, etc. (Fig 3C and 3D). CC were mainly enriched in membrane microdomain, high-density lipoprotein particle, blood microparticle, nuclear transcription factor complex, and RNA polymerase II transcription factor complex, etc. (Fig 3E and 3F).

**Fig 4:**
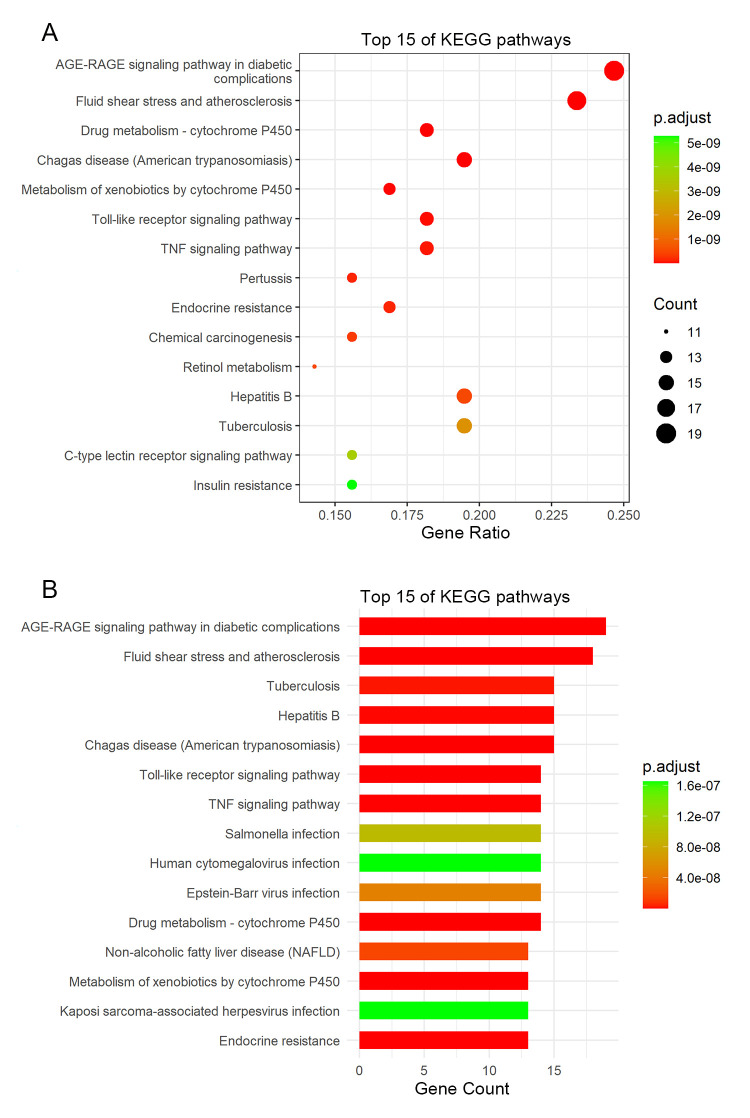
Kyoto Encyclopedia of Genes and Genomes (KEGG) enrichment analysis for 83 potential targets of SHCZF against cholestasis. **(A)** The bubble chart of top 15 KEGG pathways. **(B)** The histogram of top 15 KEGG pathways.

**Fig 5:**
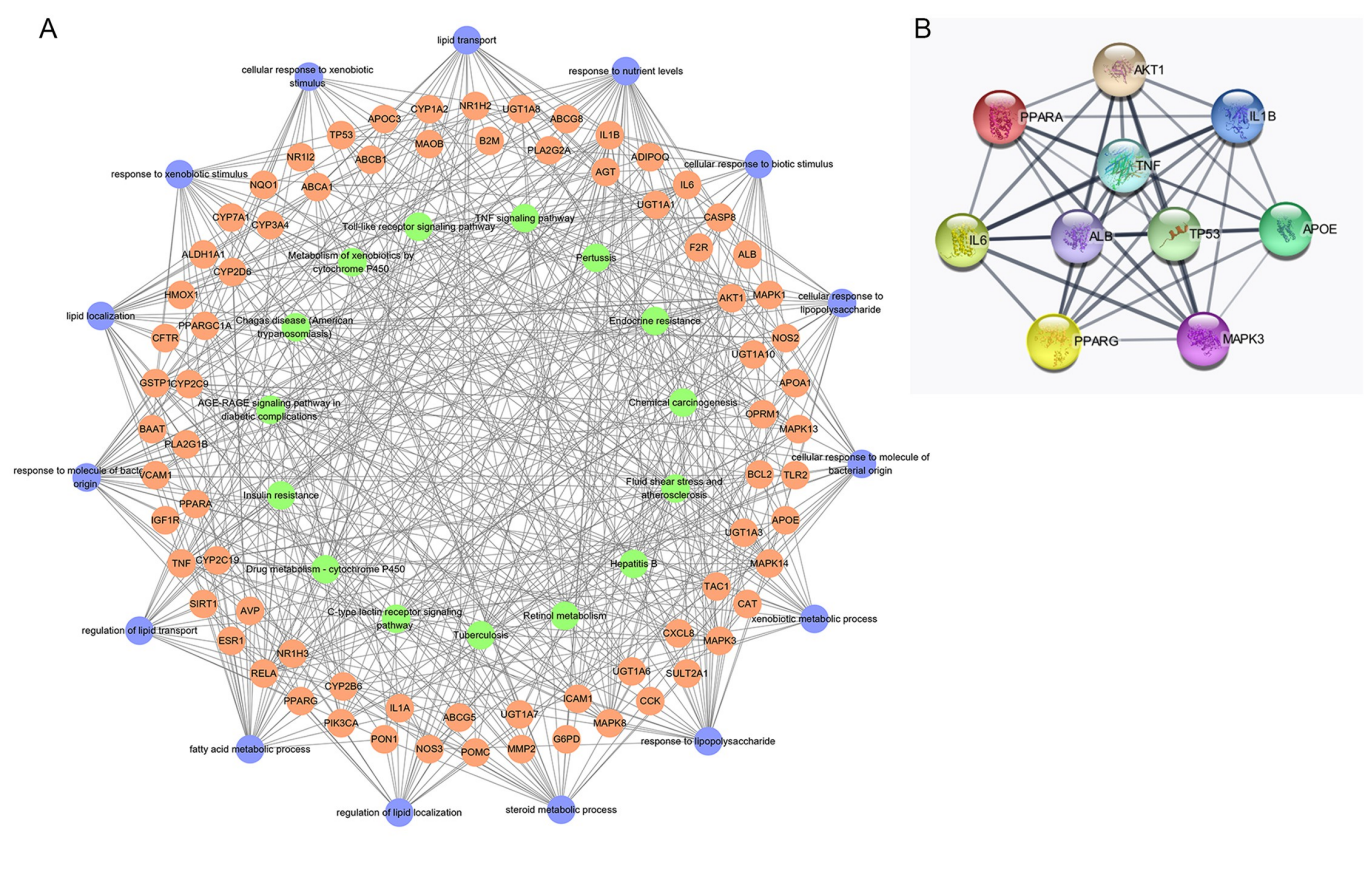
BP-target-pathway network and PPI network of top 10 hub genes for SHCZF against cholestasis. **(A)** The BP-target-pathway network included 83 potential targets (flesh-colored circles), top 15 BP terms (purple circles), and top 15 KEGG pathways (green circles). **(B)** PPI network of top 10 hub targets (ALB, IL6, AKT1, TP53, TNF, MAPK3, APOE, IL1B, PPARG, and PPARA) for SHCZF against cholestasis.

The essential signaling pathways of SHCZF in cholestasis were displayed by KEGG pathway enrichment analysis. A total of 133 pathways were significantly associated with 83 potential targets (*p* value < 0.01). In addition, the top 15 pathways with low adjust *p* values and high counts were displayed by the bubble chart and the histogram (Fig 4A and 4B), and listed in Table 4. The results showed that the common signaling pathways mainly focused on the AGE-RAGE signaling pathway in diabetic complications, Toll-like receptor signaling pathway, and TNF signaling pathway, etc. (Fig 4A and 4B). In addition, the interactions among 83 potential targets, top 15 BP terms, and top 15 pathways were visualized by a BP-target-pathway network (Fig 5A). Furthermore, the interactions among top 10 hub targets, namely ALB, IL6, AKT1, TP53, TNF, MAPK3, APOE, IL1B, PPARG, and PPARA, were visualized by a PPI network. The network showed 10 target nodes connected by 44 edges with an average degree of 8.8 (Fig 5B).

**Table 4:** Top 15 KEGG pathways for SHCZF against cholestasis.

ID	Pathway	*P*. adjust	Count
hsa04933	AGE-RAGE signaling pathway in diabetic complications	7.28E-18	19
hsa05418	Fluid shear stress and atherosclerosis	5.18E-14	18
hsa00982	Drug metabolism—cytochrome P450	2.46E-13	14
hsa05142	Chagas disease (American trypanosomiasis)	1.37E-12	15
hsa00980	Metabolism of xenobiotics by cytochrome P450	1.08E-11	13
hsa04620	Toll-like receptor signaling pathway	2.58E-11	14
hsa04668	TNF signaling pathway	6.29E-11	14
hsa05133	Pertussis	1.40E-10	12
hsa01522	Endocrine resistance	1.50E-10	13
hsa05204	Chemical carcinogenesis	3.32E-10	12
hsa05161	Hepatitis B	4.98E-10	15
hsa00830	Retinol metabolism	4.98E-10	11
hsa05152	Tuberculosis	1.93E-09	15
hsa04625	C-type lectin receptor signaling pathway	3.61E-09	12
hsa04931	Insulin resistance	5.28E-09	12

### Molecular docking between bioactive compounds and hub targets

**Fig 6:**
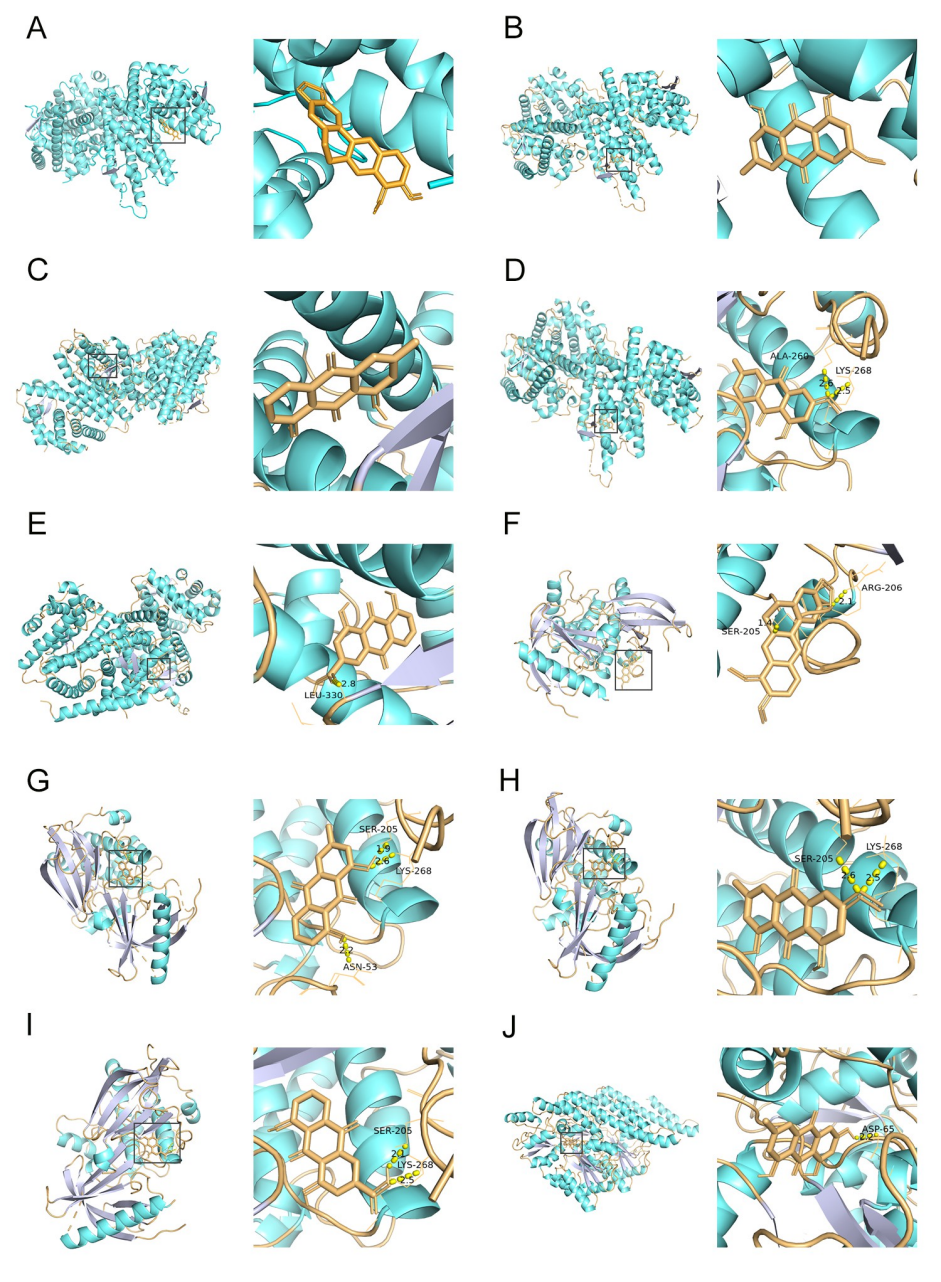
Molecular docking of SHCZF compounds and hub target proteins. **(A-E)** The binding mode of APOE and berberine chloride, physcion, chrysophanol, emodin, and rhein, respectively. **(F-I)** The binding mode of AKT1 and berberine chloride, chrysophanol, physcion, and rhein, respectively. **(J)** The binding mode of TP53 and emodin.

Molecular docking was performed to validate the interactions between bioactive compounds and hub targets of SHCZF against cholestasis. Seven main compounds of SHCZF, including chrysophanol, emodin, physcion, rhein, aloe-emodin, berberine chloride, and gardenoside, were chosen for molecular docking based on their high contents analyzed by HPLC. Top 10 hub targets, including ALB, IL6, AKT1, TP53, TNF, MAPK3, APOE, IL1B, PPARG, and PPARA, were chosen for molecular docking based on network pharmacology. The results presented that the molecular docking affinity of seven active compounds with top 10 hub target proteins were all less than -5 kcal/mol (S3 Table). The strongest binding activity between active compounds and hub target proteins were exhibited in Fig 6. APOE displayed the best binding affinity with berberine chloride (affinity = -10.5 kcal/mol), physcion (affinity = -10 kcal/mol), chrysophanol (affinity = -9.9 kcal/mol), emodin (affinity = -9.8 kcal/mol), and rhein (affinity = -9.8 kcal/mol) (Fig 6A–6E). AKT1 had a strong affinity with berberine chloride (affinity = -10.4 kcal/mol), chrysophanol (affinity = -9.7 kcal/mol), physcion (affinity = -9.7 kcal/mol), and rhein (affinity = -9.7 kcal/mol) (Fig 6F–6I). TP53 bound to emodin with a binding energy of -9.5 kcal/mol (Fig 6J). According to the molecular docking diagrams, the structures of emodin bound to sites of ALA-260 and LYS-268, while rhein interacted with LEU-330 in APOE by hydrogen bond (Fig 6D and 6E). Berberine chloride bound to sites of ARG-206 and SER-205 in AKT1, while chrysophanol bound to sites of SER-205, LYS-268, and ASN-53 (Fig 6F and 6G). Both physcion and rhein bound to sites of SER-205 and LYS-268 in AKT1 (Fig 6H and 6I). The structure of emodin bound to the site of ASP-65 in TP53 (Fig 6J).

## Discussion

Cholestasis is clinical condition and pathogenic features caused by the impairment of bile flow, which is closely associated with hepatocyte dysfunction and liver diseases [38]. Previous study indicated that SHCZF had the potential for cholestasis treatment, however, the pharmacological mechanisms remain unclear [6]. Our study found that SHCZF possessed 7 major chemical compounds, including chrysophanol, emodin, physcion, rhein, aloe-emodin, berberine chloride, and gardenoside. According to the network pharmacology analysis, 141 bioactive compounds and 83 potential targets of SHCZF against cholestasis were screened. The corresponding biological functions of potential targets were characterized and presented by Go terms and KEGG pathways. Furthermore, the interactions between 7 major bioactive compounds and top 10 hub target proteins were exhibited by molecular docking.

SHCZF is a Chinese medicine formula, presenting a hepatoprotective effect on intrahepatic cholestasis [6]. There are five herbs in SHCZF, including Dahuang (*Rhei Radix Et Rhizome*), Huangbai (*Phellodendri Chinrnsis Cortex*), Huangzhizi (*Gardeniae Fructus*), Chaihu (*Radix Bupleuri*) and Baizhu (*Atractylodes Macrocephala Koidz*.). Our study identified 7 major chemical compounds in five herbs of SHCZF, including chrysophanol, emodin, physcion, rhein, aloe-emodin, berberine chloride, and gardenoside. Previous studies indicated that these seven compounds have favorable pharmacological properties including anticancer, hepatoprotective, anti-inflammatory, etc. [39–45]. For instance, emodin can suppress liver injury and bile acids secretion, and exert a protective effect on intrahepatic cholestasis [40]. Physcion is a novel liver protective agent by reprogramming the hepatic circadian clock [41]. Rhein may promote bile acid transport and reduce bile acid accumulation in liver [42]. As a result, we speculate that these seven compounds from SHCZF may exert critical effects for SHCZF against cholestasis.

Network pharmacology are widely applied in elucidating the biological mechanism of traditional Chinese medicine formula by constructing intricate interaction network based on bioactive compounds, targets, and biological functions [46]. According to the network pharmacology analysis, a total of 141 bioactive compounds and 83 potential targets of SHCZF against cholestasis were collected based on public databases. The interactions among 83 targets were presented by a PPI network containing 83 target nodes connected by 1034 edges with an average node degree of 24.9. Besides, the interactions between 141 bioactive compounds and 83 potential targets were visualized by a C-T network. The top 10 hub targets were ALB, IL6, AKT1, TP53, TNF, MAPK3, APOE, IL1B, PPARG, and PPARA. Of note, most of them is associated with the progression of liver diseases [47–51]. For instance, ALB is a protein produced by liver, which is widely used as a marker for liver diseases [47]. IL6 and TNF are inflammatory biomarkers for cholestatic liver injury [48]. AKT1 and TP53 are closely related to the regulation of liver cancer progression [50, 51]. These results suggest that these top 10 hub targets may act as essential roles in SHCZF for cholestasis treatment.

In order to further investigate the underlying mechanisms, the biological functions of hub targets were enriched via GO and KEGG analyses. The interactions among 83 potential targets, top 15 related BP terms, and top 15 KEGG pathways were presented by a BP-target-pathway network. Our study showed that these targets were mainly related to the processes of response to molecule of bacterial origin, response to nutrient levels, response to lipopolysaccharide, etc. A previous study also found that patients with cholestasis presented a lack of response to bacterial infections [52]. These results suggested that these targets may be involved in the regulation of SHCZF against cholestasis via moderating these biological processes. In addition, the top 15 KEGG pathways related to hub targets were AGE-RAGE signaling pathway in diabetic complications, fluid shear stress and atherosclerosis, drug metabolism-cytochrome P450, TNF signaling pathway, insulin resistance, etc. According to previous statistic, the pathways of AGE-RAGE signaling pathway in diabetic complications, fluid shear stress and atherosclerosis, and insulin resistance were also enriched in non-alcoholic fatty liver and involved in the regulation of liver function [53]. Xue et al. [54] found that Da-Huang-Xiao-Shi decoction could upregulate the expression of the metabolic enzyme cytochrome P450 in chronic cholestasis. Our previous study suggested that TNF signaling pathway may be the important mechanism for SHCZF against cholestasis [6]. Overall, the above pathways may be closed relevant to SHCZF against cholestasis.

The binding force of a drug with target proteins is a pivotal index for assessing its mechanistic action on diseases [55]. The binding models between 7 SHCZF compounds and 10 hub target proteins were visualized by molecular docking. The results showed that chrysophanol, physcion, rhein, aloe-emodin, and berberine chloride had a strong affinity with APOE and AKT1. Emodin had a strong affinity with APOE, AKT1, and TP53. The structures of emodin and rhein bound to sites of SER-278 and LEU-330 in APOE, respectively. The structure of berberine chloride bound to sites of ARG-206 and SER-205 in AKT1, while chrysophanol bound to sites of SER-205, LYS-268, and ASN-53. Both physcion and rhein bound to sites of SER-205 and LYS-268 in AKT1. The structure of emodin bound to the site of ASP-65 in TP53. Differences in the binding sites may affect the ability of SHCZF compounds to bind target proteins, thereby exerting regulatory effects on cholestasis.

In conclusion, the interactions of 141 bioactive compounds and 83 potential targets of SHCZF against cholestasis were characterized by network pharmacology analysis. These targets may be closely related to the biological processes of response to molecule of bacterial origin, response to nutrient levels, response to lipopolysaccharide, etc., and involved in the pathways of AGE-RAGE signaling pathway in diabetic complications, fluid shear stress and atherosclerosis, drug metabolism-cytochrome P450, TNF signaling pathway, insulin resistance, etc. Molecular docking validated the binding of 7 active compounds and top 10 hub target proteins. Chrysophanol, physcion, rhein, aloe-emodin, and berberine chloride had a strong affinity with APOE and AKT1, and emodin had a strong affinity with APOE, AKT1, and TP53. This study provides essential clues to further explore the underlying mechanisms of SHCZF against cholestasis. However, *in vivo* or *in vitro* experiments are needed to be performed for validating the mechanisms of SHCZF against cholestasis through moderating above hub targets and pathways.

## Supporting information

S1 Fig High Performance Liquid Chromatography (HPLC) chromatograms of 7 major chemical compounds in SHCZF. **(A)** Chrysophanol. **(B)** Emodin. **(C)** Physcion. **(D)** Rhein. **(E)** Aloe-emodin. **(F)** Berberine chloride. **(G)** Gardenoside.
(PDF)

S1 Table Cholestasis-related targets from public databases. (XLSX)

S2 Table 162 active compounds and 457 corresponding targets of SHCZF. (XLSX)

S3 Table Molecular docking of seven bioactive compounds and top 10 targets. (DOCX)
