# Supplementary figures and images for: Network pharmacology analysis and molecular docking to unveil the potential mechanisms of San-Huang-Chai-Zhu formula treating cholestasis
Source: PLoS One. 2022 Feb 23;17(2):e0264398. doi: 10.1371/journal.pone.0264398 (PMC8865668; doi:10.1371/journal.pone.0264398)

**(A)** Chrysophanol

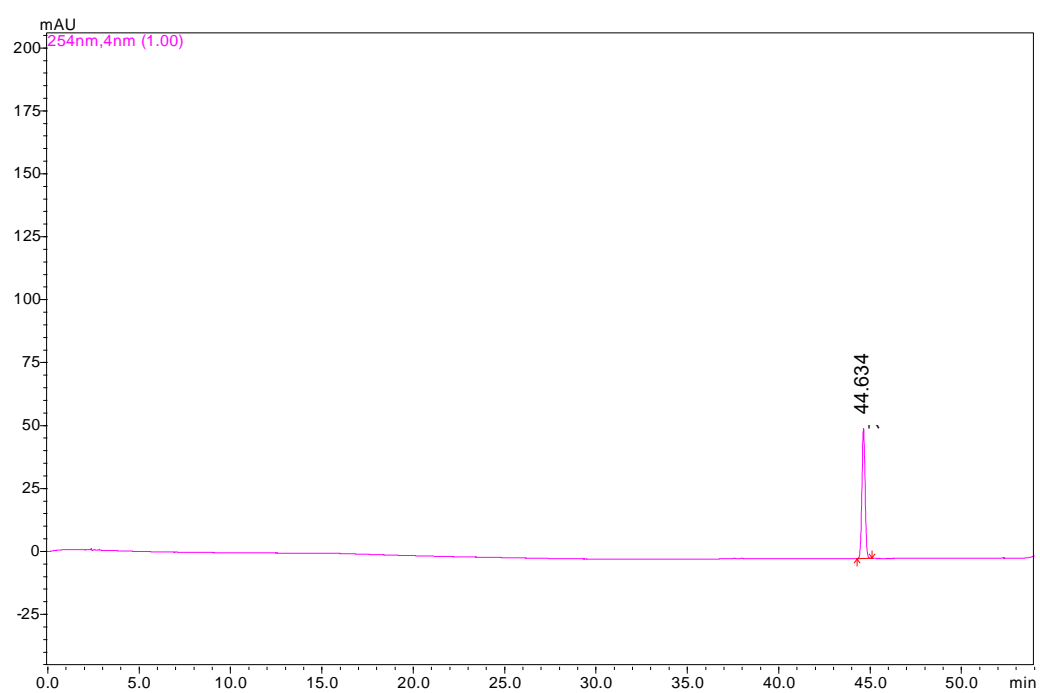

**(B)** Emodin

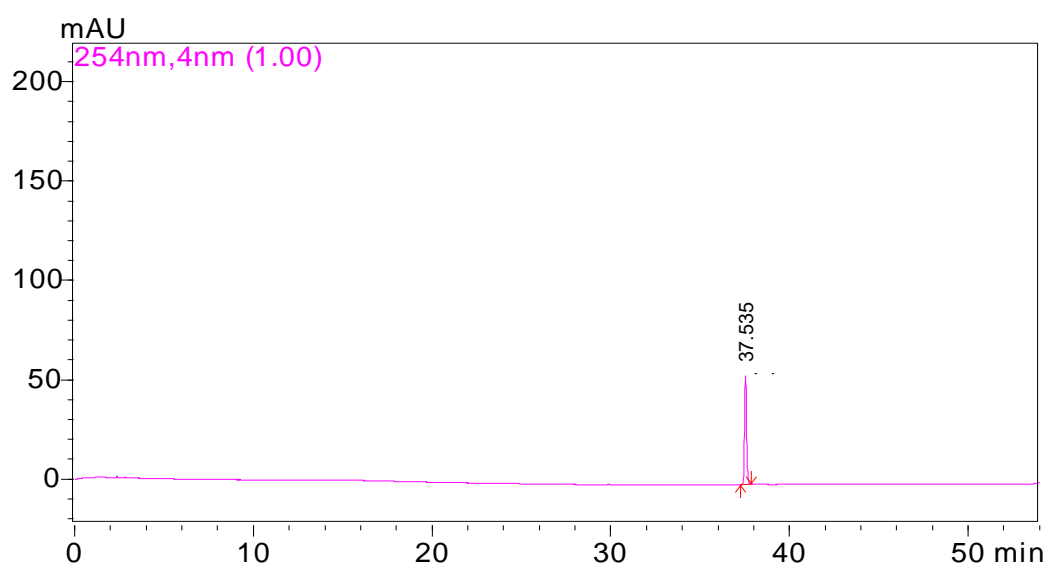

### (C) Phycion

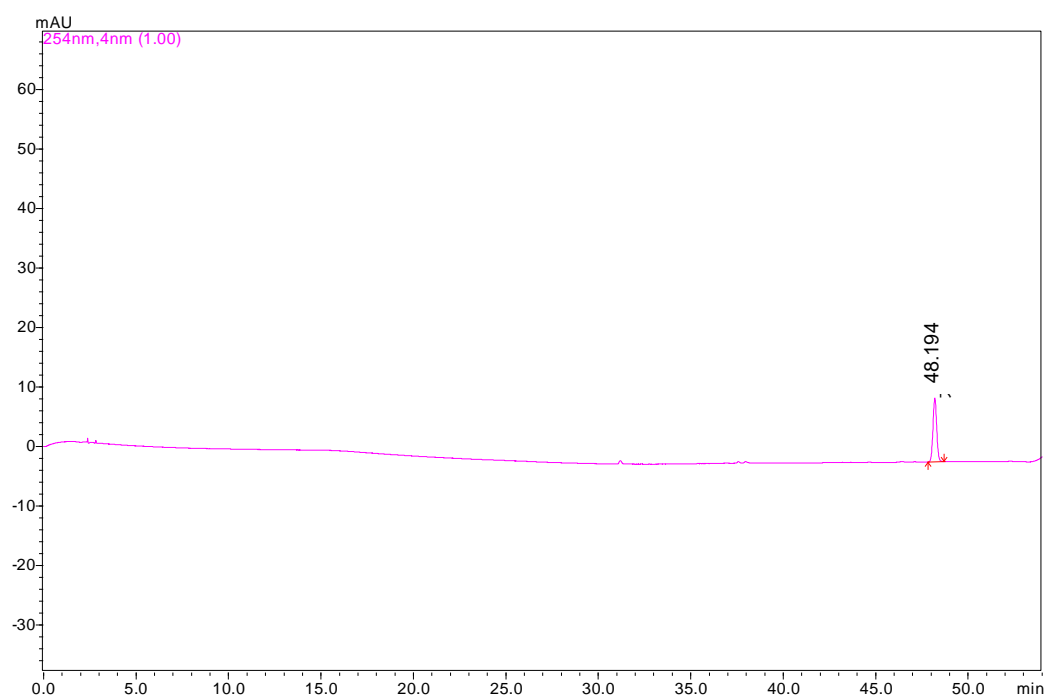

### (D) Rhein

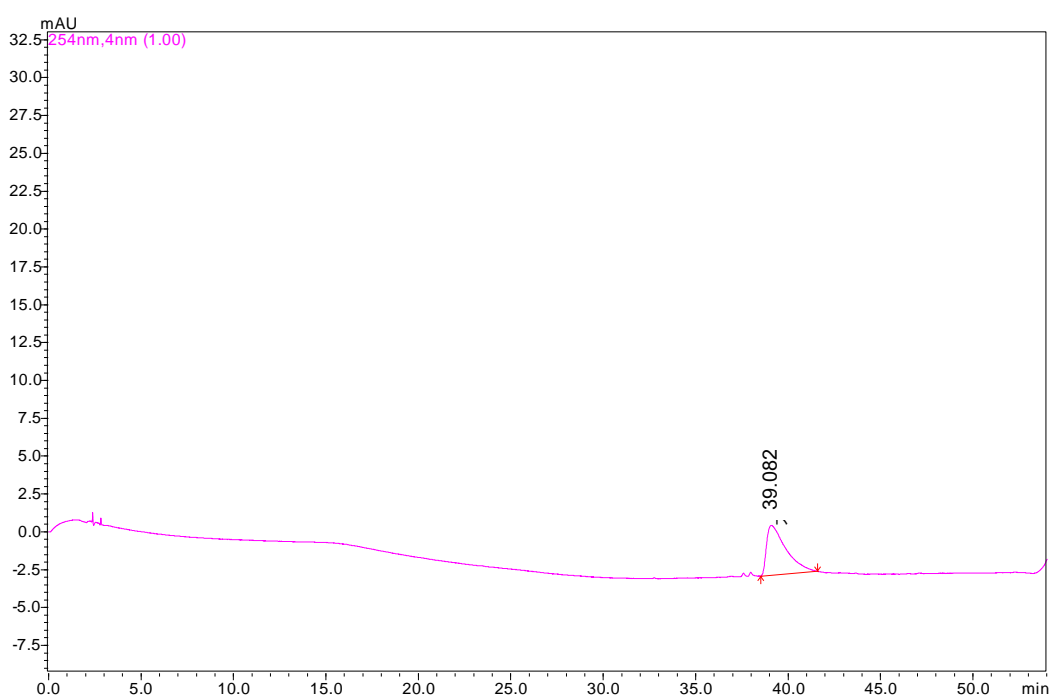

**(E) Aloe-emodin**

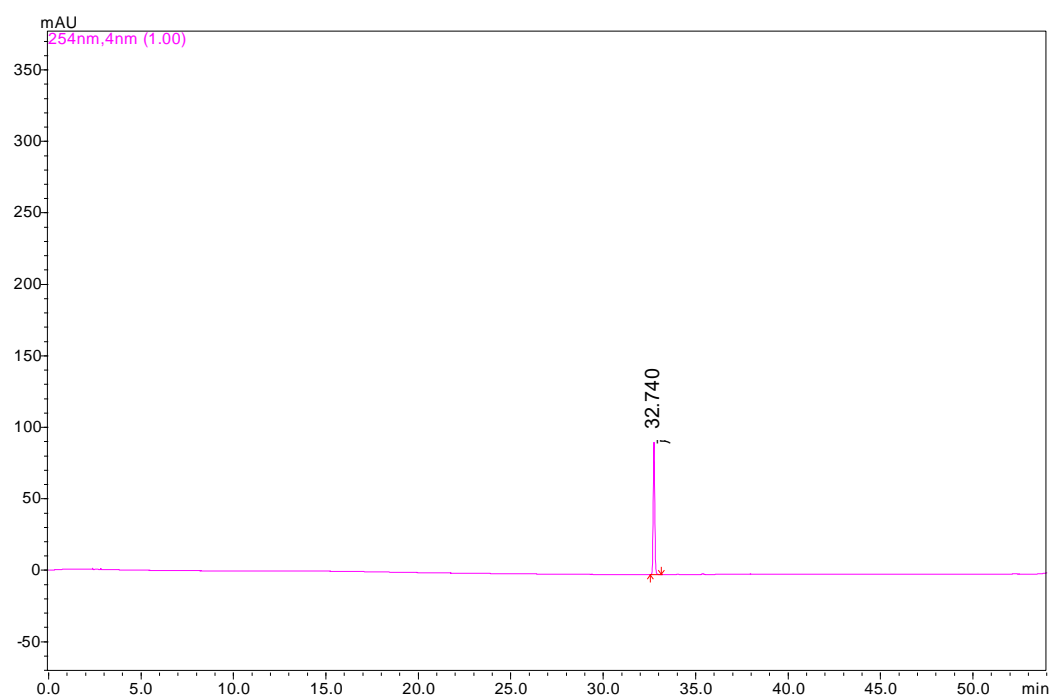

**(F) Berberine chloride**

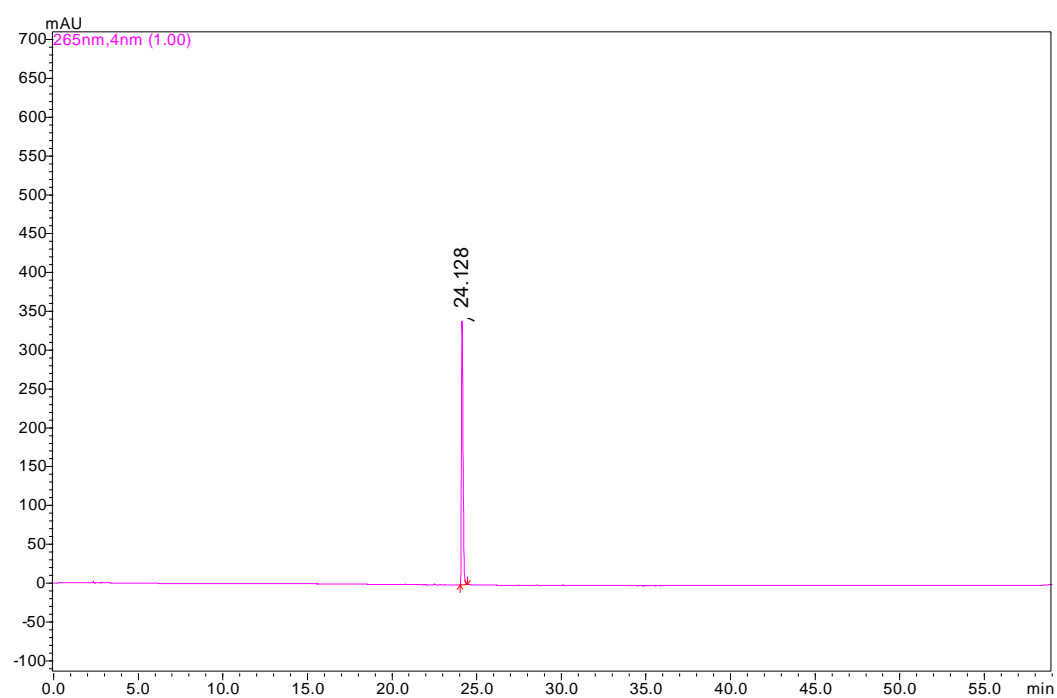

**(G)** Gardenoside

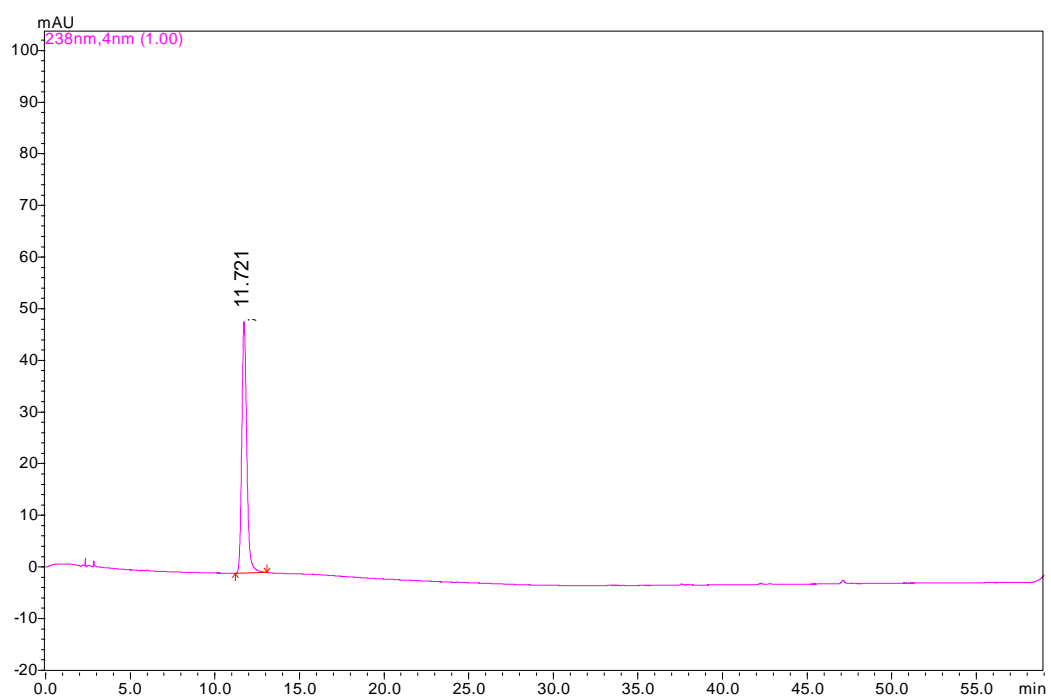

Supplement: S1 Fig — (A) Chrysophanol. (B) Emodin. (C) Physcion. (D) Rhein. (E) Aloe-emodin. (F) Berberine chloride. (G) Gardenoside. (PDF) [file pone.0264398.s001.pdf]
