# Supplementary material for: Network pharmacology analysis and molecular docking to unveil the potential mechanisms of San-Huang-Chai-Zhu formula treating cholestasis
Source: PLoS One. 2022 Feb 23;17(2):e0264398. doi: 10.1371/journal.pone.0264398 (PMC8865668; doi:10.1371/journal.pone.0264398)
Supplement: S3 Table — (DOCX) [file pone.0264398.s004.docx]

**Table S3 Molecular docking of seven bioactive compounds and top 10 targets.**

| **Chem** | **PDB** | **GENE** | **Best affinity** |
| --- | --- | --- | --- |
| Berberine Chloride | APOE | 5hjp | -10.5 |
| Berberine Chloride | AKT1 | 3o96 | -10.4 |
| Physcion | APOE | 5hjp | -10 |
| Chrysophanol | APOE | 5hjp | -9.9 |
| Emodin | APOE | 5hjp | -9.8 |
| Rhein | APOE | 5hjp | -9.8 |
| Chrysophanol | AKT1 | 3o96 | -9.7 |
| Physcion | AKT1 | 3o96 | -9.7 |
| Rhein | AKT1 | 3o96 | -9.7 |
| Emodin | TP53 | 4xzs | -9.5 |
| Emodin | AKT1 | 3o96 | -9.5 |
| Aloe-Emodin | APOE | 5hjp | -9.4 |
| Chrysophanol | MAPK3 | 4qtb | -9.3 |
| Physcion | MAPK3 | 4qtb | -9.3 |
| Aloe-Emodin | AKT1 | 3o96 | -9.2 |
| Chrysophanol | TP53 | 4xzs | -9.2 |
| Emodin | MAPK3 | 4qtb | -9.1 |
| Rhein | MAPK3 | 4qtb | -8.9 |
| Berberine Chloride | MAPK3 | 4qtb | -8.9 |
| Aloe-Emodin | MAPK3 | 4qtb | -8.7 |
| Aloe-Emodin | TP53 | 4xzs | -8.6 |
| Berberine Chloride | PPARG | 7awd | -8.4 |
| Rhein | TP53 | 4xzs | -8.2 |
| Chrysophanol | PPARG | 7awd | -8.2 |
| Physcion | IL6 | 4qil | -8 |
| Berberine Chloride | IL6 | 4qil | -8 |
| Berberine Chloride | PPARA | 3et1 | -7.9 |
| Physcion | TP53 | 4xzs | -7.9 |
| Rhein | PPARG | 7awd | -7.9 |
| Rhein | TNF | 3m0d | -7.8 |
| Gardenoside | AKT1 | 3o96 | -7.8 |
| Chrysophanol | IL6 | 4qil | -7.8 |
| Physcion | PPARG | 7awd | -7.8 |
| Aloe-Emodin | PPARG | 7awd | -7.8 |
| Rhein | PPARA | 3et1 | -7.7 |
| Gardenoside | TNF | 3m0d | -7.7 |
| Emodin | IL6 | 4qil | -7.7 |
| Rhein | IL6 | 4qil | -7.7 |
| Gardenoside | TP53 | 4xzs | -7.7 |
| Berberine Chloride | ALB | 6rq7 | -7.7 |
| Emodin | PPARG | 7awd | -7.7 |
| Gardenoside | PPARA | 3et1 | -7.6 |
| Gardenoside | APOE | 5hjp | -7.6 |
| Physcion | PPARA | 3et1 | -7.5 |
| Aloe-Emodin | IL6 | 4qil | -7.5 |
| Aloe-Emodin | PPARA | 3et1 | -7.4 |
| Aloe-Emodin | TNF | 3m0d | -7.4 |
| Berberine Chloride | TNF | 3m0d | -7.4 |
| Gardenoside | MAPK3 | 4qtb | -7.4 |
| Physcion | TNF | 3m0d | -7.3 |
| Emodin | PPARA | 3et1 | -7.2 |
| Chrysophanol | TNF | 3m0d | -7.1 |
| Emodin | TNF | 3m0d | -7.1 |
| Chrysophanol | PPARA | 3et1 | -7 |
| Gardenoside | PPARG | 7awd | -6.8 |
| Gardenoside | IL6 | 4qil | -6 |
| Berberine Chloride | TP53 | 4xzs | -6 |
| Rhein | ALB | 6rq7 | -5.9 |
| Chrysophanol | ALB | 6rq7 | -5.7 |
| Emodin | ALB | 6rq7 | -5.6 |
| Aloe-Emodin | ALB | 6rq7 | -5.6 |
| Physcion | ALB | 6rq7 | -5.5 |
| Gardenoside | ALB | 6rq7 | -5.4 |
| Berberine Chloride | IL1B | 6y8m | -5.2 |
| Rhein | IL1B | 6y8m | -5 |
