# Supplementary material for: Network pharmacology analysis and molecular docking to unveil the potential mechanisms of San-Huang-Chai-Zhu formula treating cholestasis
Source: PLoS One. 2022 Feb 23;17(2):e0264398. doi: 10.1371/journal.pone.0264398 (PMC8865668; doi:10.1371/journal.pone.0264398)
Supplement: Attachment — Submitted filename: Response to Reviewers.docx [file pone.0264398.s005.docx]

**Response to Reviewers**

The authors would like to appreciate the editor and reviewers for their precious time and invaluable comments for our work. We have carefully revised our manuscript according to the reviewers’ comments. We also have reviewed the reference list in the manuscript and ensure that it is complete and correct. We believe that the manuscript has been further improved. The corresponding revisions and refinements made in the manuscript are summarized in our response below.

Reviewer #1: In this report, Yao et al present a very through and complex bioinformatic analysis of the molecular mechanism of a Chinese medicine formula, San-Huang-Chai-Zhu formula (SHCZF), against cholestasis. They identified 7 major active chemical compounds of SHCZF by HPLC and revealed their targets via data mining. By comparing with the cholestasis-related genes that retrieved from public databases, 83 over-lapping targets were pinned down as core targets of the SHCZF active compounds against cholestasis. Although this study didn’t provide any rigor and independent confirmation due to the scope of the research, it provided some profound insight of the underlying mechanism of SHCZF against cholestasis, which is highly novel and significant in the larger scheme of developing novel therapeutic strategy against cholestasis. I would suggest the manuscript be accepted in the current form with some minor modifications such as:

**Q:** Define the abbreviations at the first use for the less initiated, such as: GO=Gene Ontology; CC=Cellular Component.

**R:** We thank the reviewer for pointing this out. We have checked the full article and defined all abbreviations at the first use in the manuscript.

**Q:** Detailed results description are strongly advised for a better understanding of the analysis. For example, in molecular docking analysis, the authors should provide some more details about the interactions between the active compounds and their target molecules.

**R:** Thank you for this professional suggestion. We have added more details in the manuscript about molecular docking, including the binding affinity and sites between active compounds and hub target proteins.

Reviewer #2: The manuscript submitted by Liu et al used network pharmacology and molecular docking approach to identify the targets of key ingredients/ compounds found in the formulated Chinese medicine SHCZF. The data and methods presented are clear and detailed. Some minor comments to be addressed before it is considered for publication.

**Q:** 1. Figure 5 and 6 can be part of the same figure.
**R:** We agree the reviewer’s suggestion and have merged Figure 5 and 6 into the same figure (revised Fig. 5).

**Q:** 2. Table S1 presents critical data and should become part of the main manuscript.

**R:** Although we tend to agree the reviewer, the data in the Table S1 are too large to be included in the main manuscript. In addition, the 83 potential targets screened from the data of Table S1 are more important and have been included in the main manuscript. Thus, we provided Table S1 as a supplementary material.

**Q:** 3. Figure 7 needs some more annotation/labelling. Apart from the amino acids, the interacting molecules are not annotated in the figure.

**R:** Thanks for pointing this out. We have added more annotations in Fig. 6 (Fig. 7 was changed to Fig. 6), including interacting molecules by hydrogen bonds.

**Q:** 4. Please proof-read for minor drafting issues.

**R:** We have checked the full article and revised drafting issues with track changes in the manuscript.
